# Intergenerational educational mobility and mental health: Evidence from a Filipino birth cohort

**DOI:** 10.1371/journal.pgph.0004570

**Published:** 2025-08-12

**Authors:** Lucy Barrass, Maria Theresa Redaniel, Lucy Riglin, Nanette R. Lee, Laura D. Howe, Duleeka Knipe

**Affiliations:** 1 Population Health Sciences, Bristol Medical School, University of Bristol, Bristol, United Kingdom; 2 NIHR ARC West, Population Health Sciences, University of Bristol, Bristol, United Kingdom; 3 National Cancer Registry of Ireland, Cork, Ireland; 4 Division of Psychological Medicine and Clinical Neurosciences, The Wolfson Centre for Young People’s Mental Health and, Centre for Neuropsychiatric Genetics and Genomics, Cardiff University, Cardiff, United Kingdom; 5 USC-Office of Population Studies Foundation, Inc., University of San Carlos, Cebu City, Philippines; 6 South Asian Clinical Toxicology Research Collaboration, Faculty of Medicine, University of Peradeniya, Peradeniya, Sri Lanka; 7 NIHR Bristol Biomedical Research Centre, University Hospitals Bristol and Weston NHS Foundation Trust and University of Bristol, Bristol, England; Indiana University Richard M Fairbanks School of Public Health, UNITED STATES OF AMERICA

## Abstract

Evidence suggests that associations between educational attainment and mental health may vary according to educational mobility (i.e., changes in educational attainment across generations). This evidence is largely limited to high-income countries. Using data from an ongoing birth cohort (Cebu Longitudinal Health and Nutrition Survey), we assessed the associations with mental health for both own educational attainment and intergenerational educational mobility (i.e., the difference in educational attainment between parents during pregnancy and the offspring aged 18 and 35 years) in 2,038 Filipino individuals. Primary mental health outcomes were depressive symptoms, suicidal ideation and psychological distress at age 35 years, with the former two measures used as secondary outcomes at age 18 years. We used logistic regression models, adjusting for sex and urbanicity at birth. Lower levels of educational attainment at age 35 years were associated with higher odds of depressive symptoms (Odds Ratio (OR): 1.68; 95% CI: 1.11-2.52) and psychological distress (OR: 1.54; 95% CI: 1.09-2.17), but confidence intervals for suicidal ideation crossed the null (OR: 1.43; 95% CI: 0.91-2.26). Participants who had lower educational attainment than their parents at age 35 years (downward educational mobility) had higher odds of depressive symptoms compared to participants where both they and their parents had higher levels of educational attainment (OR: 3.12; 95% CI: 1.46-6.66). There was no statistical evidence of this association for the other outcomes. We also did not find statistical evidence that upward educational mobility was associated with mental health outcomes. Filipino individuals who had lower educational attainment than their parents showed higher levels of depressive symptoms compared to those whose parents and own education were both in the higher education category. We found limited evidence of these associations for other measured mental health outcomes.

## Introduction

Mental health disorders currently represent a significant and increasing public health concern, especially in low- and middle- income countries (LMICs) [1,2]. They are a leading cause of disease burden worldwide. Research has shown that lower socioeconomic position (SEP) is associated with a higher risk of worse mental health [3–6]. Education is a commonly used indicator of SEP and associations between lower SEP and worse health may be due to lower levels of education preventing individuals from being able to access health services and from individuals being less likely to invest (e.g., time) in healthy behaviours due to decreased receptiveness to health education measures [7]. Likewise, higher levels of education can lead to hopefulness, better self-efficacy and a sense of control through greater expectations for the future, which is particularly relevant for mental health [8]. Additionally, education is a key determinant of employment opportunities and therefore, income, wealth, housing and most other SEP indicators [9].

Social mobility, which refers to the changes in SEP either between generations (intergenerational), or within individuals (intragenerational), may also be associated with mental health [10]. Social mobility is often measured using indicators of SEP, such as income (income mobility), occupation (occupational mobility) or education (educational mobility), either individually or in a composite measure [10]. Whilst relationships between current SEP and health are more widely studied, little is known about social mobility and its potential health outcomes, particularly mental health, in LMICs. Whilst existing cross-sectional research can provide information about associations with SEP circumstances, it does not account for prior life-course SEP trajectories that may be influencing present outcomes.

A recent systematic review highlighted the importance of considering social mobility, measured via single and composite measures of SEP, when examining associations with mental health [11]. When compared to those who had stable high SEP, upward or downward mobility were associated with more mental health problems, whereas when compared to stable low SEP, upward or downward social mobility was associated with fewer mental health problems. Downward mobility was associated with more mental health problems when compared to upward mobility [11]. Elevated rates of worse mental health in the upwardly mobile group in comparison to the stable high SEP group could reflect the feeling of dissociation from their old social standing and the feeling of not belonging within their new group, or the longer lasting effects of their SEP in early life [12–15]. The abovementioned review identified 21 studies, with only two from LMICs outside of Europe [16,17] and none from countries in Southeast Asia. This suggests a lack of information about the effect of social mobility on mental health in this geographic area, as well as in LMICs. In addition, the effects of lower SEP in LMICs are likely to be more profound than in high-income settings which may underestimate the influence of remaining in this SEP position throughout the life course in LMICs.

There is also currently very little evidence on the association between social mobility and suicidal ideation. As there are close links between suicidal ideation and suicide, and the latter is a leading cause of mortality globally, evidence is needed on the links to prevent suicidal ideation [18]. The one paper identified on this topic suggests that remaining in stable low SEP or being upwardly or downwardly mobile was associated with higher odds of suicidal ideation compared to remaining in stable high when SEP was measured through dwelling status [19]. When considering financial status, levels of suicidal ideation were found to be similar in stable high and upwardly mobile groups. With an increasing burden of mental health worldwide, determining the role of parental SEP in mental health outcomes in later life, could aid targeted interventions for at-risk groups.

Some measures used in high-income studies to assess social mobility are not appropriate in LMICs e.g. working status or social class, due to the importance of informal economies and seasonal nature of some employment, and therefore, findings from these studies may not be applicable in LMIC settings [20]. Education is one of the most used measures of SEP in epidemiological research and is particularly useful in LMICs where the unpredictability of income and different occupational structures make other measures of SEP more difficult to study [10,20]. Education is useful to explore when interested in mobility as it rarely changes after early adulthood, is not subject to the stigma some other measures face and is not prone to high levels of recall bias. Comparing educational levels across generations is often simpler and more practical than changes in income, occupation and wealth which can be difficult to measure in equivalent ways across two generations due to time trends in employment characteristics and availability of goods typically included in a wealth index. Three papers from the systematic review on this topic explored the association between intergenerational educational mobility, i.e., changes in educational attainment between generations, and mental health [11,21–23].

Using data from the Cebu Longitudinal Health and Nutrition Survey (CLHNS), we aimed to assess if and how educational mobility was associated with depression, suicidal ideation and psychological distress at two different life stages.

## Methods

### Ethics statement

Ethical approval was not obtained for the secondary analysis of this data, however, all surveys and data protections were carried out in accordance with The Code of Ethics of the Declaration of Helsinki, with IRB approvals for initial data collection obtained from the University of North Carolina at Chapel Hill, and the University of San Carlos Research Ethics Committee, Cebu, Philippines. IRBs reviewed and approved the conduct of the original data collection study, including its overall protocol, instruments and informed consent process. They also covered data access to other interested parties under appropriate assurances of procedures to ensure participant privacy and data security. Participants provided written consent.

### Study setting

Situated in Southeast Asia, the Philippines is classified by the World Bank as a lower-middle income country with a GDP per capita of US$3,498.5 [24]; of the 11 countries in South East Asia, the Philippines ranks 7^th^ on this measure. Metropolitan Cebu (population just under 3.2 million) is one of the largest metropolitan areas in the Philippines, with high density neighbourhoods, less dense peri-urban areas, rural towns and more isolated communities in the mountains and islands [25,26]. It is located in the Visayas (one of three principal island groups in the Philippines) and currently comprises seven cities and six municipalities, which are different categories of local government units determined by population size and income.

### Data

Data were from the CLHNS, a birth cohort initiated in 1983 collecting data from areas within Metropolitan Cebu. A single stage cluster sampling procedure was used to randomly select 33 of the 270 barangays, the smallest local level of government in the Philippines (17 urban, 16 rural), from across the study area. Pregnant women residing in these barangays were recruited for the study in 1983 and those who gave birth between 1 May 1983, and 30 April 1984 were included in the sample; less than 4% did not agree to participate. In their sixth or seventh month of pregnancy (baseline), 3,327 women were interviewed, of which 3,080 had a single live birth and were still eligible and willing for inclusion. The infants were a representative sample of the births in metro Cebu in the one year period [26]. CLHNS has been described in more detail elsewhere [26]. Initially, the infants and their mothers were followed up every two months for a two-year period, and then at different time points since, following the offspring through childhood and now adulthood. The latest follow up occurred in 2018, when the offspring were aged 35 years. Surveys were administered in the local language, Cebuano.

### Measures

#### Outcomes.

The outcomes were primarily studied when the offspring were aged 35 years, with a secondary analysis of outcomes at age 18 years. Depressive symptoms were measured using an adapted version of the Centre for Epidemiological Studies Depression Scale (CES-D) [27]. The adapted CES-D tool of 16 questions asked about feelings and problems in the preceding four weeks ranging from having poor digestion to worthlessness and suicidal ideation. Positive items asked included “felt happy”, “felt hopeful about the future” and “enjoyed normal daily activities”. Respondents answered on a three-point scale of either “none of the time”, “occasionally”, or “most of the time”. Each response was given a score (0–2), with positively framed questions scored in reverse. Scores were summed and a cut off was used to determine higher and lower depressive symptoms. The cut off was derived using a data driven approach, using the mean and one standard deviation above it, an approach which has been used in other studies, including in the Philippines [28,29]. At age 18 years (in 2002), this resulted in higher depressive symptoms when participants scored 12 and above, whilst it was 13 and above for age 35 years in 2018. The 16-item CES-D has not been validated in the Filipino population, however, other versions have been validated in other populations in this region [30,31].

Suicidal ideation was measured at both time points using questions from the CES-D scale. Participants were asked the following questions: “In the past 4 weeks, did you wish you were dead?”; “In the past 4 weeks, did you have the idea of taking your own life?”. Experiencing suicidal ideation was coded as yes if participants answered ‘occasionally’ or ‘most of the time’ to either of the two questions.

Psychological distress was assessed using the Self Reporting Questionnaire-20 (SRQ-20). The 20-item instrument, a screening tool recommended for use in LMIC settings, has been validated in many countries, including the Philippines, and is used commonly in Southeast Asian settings [32–37]. Participants responded either “yes” or “no” to questions about how they felt in the last month, e.g., “do you feel nervous, tense or worried?”, “Is your appetite poor?”. Scores were summed, with a response of “yes” equalling one. We used a cut-off score of 7 or higher to determine presence of psychological distress, although other studies in Southeast Asia suggest a range of cut-off points that differ from ours. We use this cut-off as it has previously been validated in the Philippines and recommended in the community setting in Vietnam, another Southeast Asian country [38–40]. This measure was not available in the age 18 years questionnaire.

#### Exposures.

We used the highest educational attainment of either parent, at baseline, as our measure of parental education. We used baseline measurements as it provided a measure of initial socioeconomic conditions. For own education, educational attainment of themselves at each age (18 and 35 years) was used. For each measure of education, we categorised participants into high education (completed high school education or higher – typically at age 16/17 years), and low education (did not complete high school). During schooling of these participants, completion of high school was not mandatory. We then classified participants into one of four educational mobility categories: 1) stable high (high parental education and high own education); 2) upward mobility (low parental education and high own education); 3) downward mobility (high parental education and low own education); 4) stable low (low parental education and low own education). One participant was excluded due to reporting not having a traditional school education when asked at age 35 years, and were excluded from the 2002 (age 18 years) analysis as it was likely this would also apply then, despite not being reported at the time.

#### Covariates.

Sex, measured at birth, was included as a binary variable. Whether an individual lived in a rural or urban area was also measured at birth. We do not adjust for variables after birth as they may mediate the relationship, not confound it, nor do we include other socioeconomic variables at birth or baseline due to the potential for overadjustment of the associations [41].

### Data analysis

Descriptive statistics were summarised using means and standard deviations for continuous measures and counts and percentages for categorical variables. Our primary analysis focused on the age 35 years data where it was likely participants had reached their final educational attainment. We included age 18 years as a secondary analysis as a higher prevalence of mental health disorders are often seen in adolescents than adulthood. The earlier age was not the focus of our analysis as participants may not yet have reached their potential educational attainment which may bias results.

We used logistic regression to estimate odds ratios (OR) and 95% confidence intervals (CI). First, we estimated crude and adjusted (for sex and urbanicity) associations between parental and own education and depressive symptoms at age 35 years. Second, we estimated crude and adjusted associations between educational mobility and each outcome. Third, as a secondary analysis, we repeated the above for exposure and outcome measures at age 18 years (Table A, B, C, D in S2 Text). Post-hoc, using logistic regression, we conducted an additional analysis that used downward mobility as our reference category, allowing us to determine whether this group had statistically different odds of mental health to stable low at age 35 years. Analyses were carried out using STATA v18.

Multiple imputation using chained equations (MICE) was used to impute missing exposure and outcome data, under the assumption that the data were missing at random. Participants were included in the imputation if they had at least one measure of mental health at either time point. Participants lost to follow-up before age 18 years were not included, unless they responded in the age 35 years follow up. Missingness ranged from 35.0% to 41.2%.

Auxiliary variables from the CLHNS cohort were used to assist the imputation. These included the variables used in the main analysis (e.g., sex and urbanicity), as well as other measures related to the outcome (e.g., depressive symptom and suicidal ideation measures at age 22), and to the exposure (e.g., earlier education measures). Educational attainment and all outcomes were imputed using logistic regression. All variables were imputed in the form they were used in the substantive analysis. A single imputation model was used to impute missing data on all outcomes and exposures. There were 75 datasets imputed with a burn-in of 60 iterations. We did not impute data for those who had been lost to follow-up before 2002 due to no prior variables being collected on mental health, and therefore, a lack of auxiliary variables used to predict mental health. The imputed dataset was used for the primary analysis and a complete case sensitivity analysis was also completed.

## Results

### Sample characteristics

**Fig 1:**
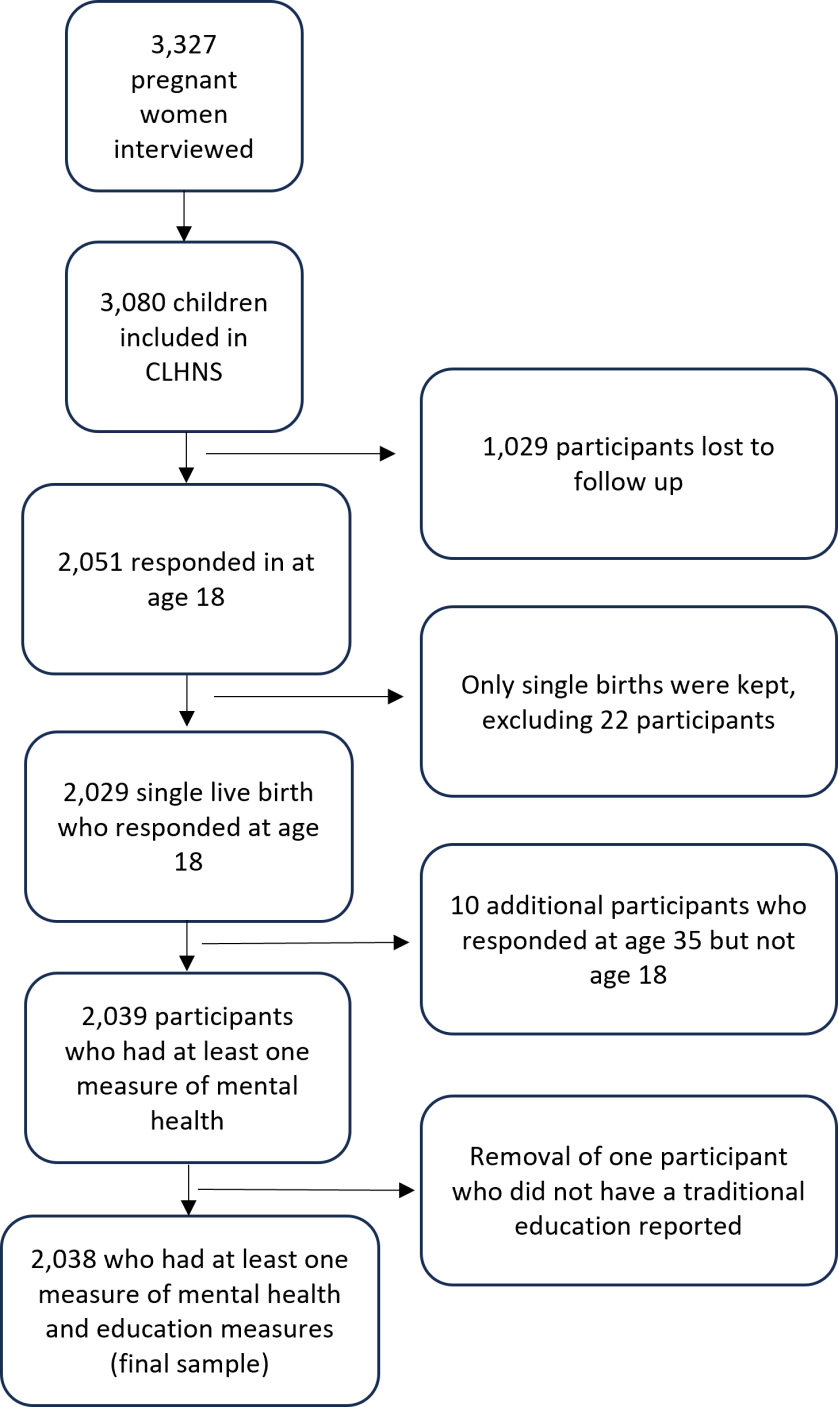
Flow chart of inclusion into the study all ages in years.

A flow chart of participant inclusion in the study can be seen in Fig 1 and characteristics of the study sample can be seen in Table 1. In total, 2,038 participants were included after multiple imputation, with 2,020 complete cases at age 18 years, and 1,193 at age 35 years. Ten participants who did not participate in the survey at age 18 years responded at age 35 years. Sample characteristics of the complete case analyses can be found in Table A in S1 Text.

**Table 1:** Summary statistics at age 35 years (2018).

Variable	(%)* n = 2038
**Male**	**53.0**
**Urban**	**73.8**
**Own education**	
High	72.6
Low	27.4
**Parental education**	
High	34.4
Low	65.6
**Mobility**	
Stable high	30.5
Upward	42.1
Stable low	23.5
Downward	3.9
**High depressive symptoms**	8.2
**Suicidal ideation**	7.4
**Psychological distress**	13.2

*Figures shown for age 35 years (2018) imputation.

High education is categorised as completed high school.

In the imputed dataset, 53.0% of participants were male, and 73.8% lived in an area classified as urban. The prevalence of each mental health outcome at age 35 years was 8.2% (95% CI: 6.5%, 9.9%), 7.4% (95% CI: 5.9%, 9.0%) and 13.2% (95% CI: 11.3%, 15.0%) for depressive symptoms, suicidal ideation and psychological distress, respectively. Results were consistent across the imputed and complete case datasets (Table A in S1 Text).

### Associations between education at birth (parental) and at age 35 years (own) and mental health at age 35 years

**Fig 2:**
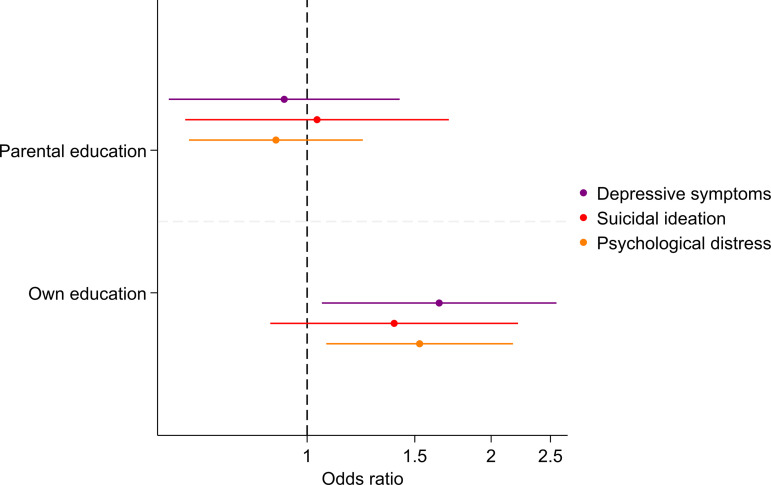
Adjusted odds ratios of the association between low education levels and mental health outcomes at age 35 years. Dashed line represents no association.

We found an association between lower levels of own education and higher odds of depressive symptoms (OR: 1.64, 95% CI: 1.06, 2.56) and psychological distress (OR: 1.53, 95% CI: 1.07, 2.17), after adjustment for sex and urbanicity at birth. There was no evidence of an elevated rate of suicidal ideation in those with lower levels of education (OR 1.39, 95% CI: 0.87, 2.21). There was very little evidence of an association between parental education and any mental health outcome at age 35 years (Fig 2). Results were consistent across the imputed and complete case datasets (measured via overlapping confidence intervals) for each time point (Table A in S3 Text).

### Associations between educational mobility and mental health at age 35 years

**Fig 3:**
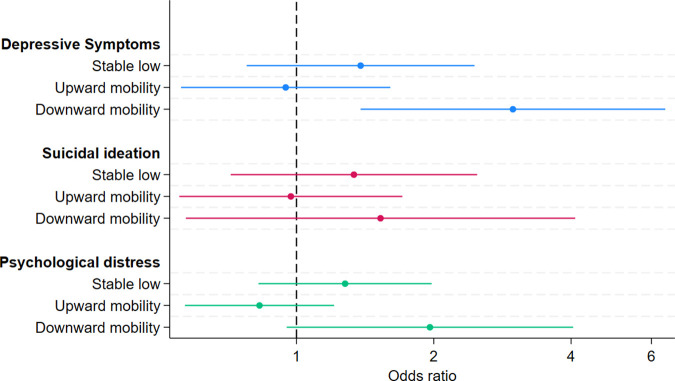
Adjusted associations between mobility and mental health outcomes at age 35 years (2018).

Our analysis found evidence of an association between downward mobility and higher odds of depressive symptoms at age 35 years (Fig 3. OR: 2.98, 95% CI: 1.38, 6.44), compared to stable high education, after adjustment for covariates. We did not find strong evidence of an association between any other category of mobility for any of the three mental health outcomes. There was some evidence of an association between downward mobility and psychological distress in the complete case analysis (OR: 2.22, 95% CI: 1.03, 4.77). Otherwise, results were similar between the imputed and complete case datasets (measured via overlapping confidence intervals) for each time point (Table B in S3 Text). Our post-hoc analysis demonstrated that, compared to stable low, those experiencing downward mobility had 2.6 times the odds of experiencing depressive symptoms (95% CI: 1.02, 4.55). There was little evidence of an association of differing odds with downward mobility, compared to stable low, with suicidal ideation (OR: 1.14; 95% CI: 0.42, 3.08) or psychological distress (OR: 1.53; 95% CI: 0.74, 3.20).

### Comparisons between age 18 and 35 years

Lower levels of individual education remained strongly associated with higher odds of depressive symptoms (OR: 2.25, 95% CI: 1.73, 2.92) and suicidal ideation (OR: 1.51, 95% CI: 1.18, 1.94) at age 18 years, after adjustment for covariates, which is broadly consistent with age 35 years findings. Psychological distress was not measured at age 18 years so there were no comparisons for these measures. The associations of lower parental education and depressive symptoms at age 18 years were consistent with age 35 years, however, there was statistical evidence of an association only at age 18 years (OR: 1.50, 95% CI: 1.13, 1.99), when compared to higher parental education.

For the educational mobility analysis, at age 18 years, we found evidence of an association between stable low and higher odds of both depressive symptoms (OR: 2.54, 95% CI: 1.79, 3.61) and suicidal ideation (OR: 1.65, 95% CI: 1.20, 2.28), compared to the stable high group. At age 18 and age 35 years, we found evidence of an association between downward mobility and higher odds of depressive symptoms, when compared to the stable high group.

## Discussion

This study aimed to assess if and how educational mobility was associated with depression, suicidal ideation and psychological distress at two different life stages. Our findings support an association between downward educational mobility and higher odds of depressive symptoms (compared to stable high education) in Filipino adults, but did not provide strong support for associations with other mental health outcomes. We also found that at age 18 years, remaining in lower levels of education (i.e., stable low) was associated with higher odds of both depressive symptoms and suicidal ideation, and that downward mobility was associated with higher odds of depressive symptoms.

Our findings are consistent with results from a systematic review which found that those with downward social mobility were more likely to experience worse mental health [11]. However, we only found this association for depressive symptoms, not all mental health outcomes we measured. Our findings differ from the review as they found upwardly mobile participants to also have worse mental health than those in the stable high group, whereas we found that this group had mental health no worse than those in the stable high group at age 35 years. A large proportion of our sample experienced upward mobility, making this the most common type of mobility. It is likely this trend continues nationwide and because of the collective nature, the transition between lower SEP to higher SEP may not have been as stressful and isolating, mitigating the effects seen in high-income countries of worse mental health. Equally, our results may have been impacted by low statistical power due to small numbers in each group, which could explain some differences. We found some evidence that, compared to stable-high, stable-low educational mobility was associated with higher odds of mental health problems at age 18 years but not age 35 years. Similarly, we found stronger evidence of parental education influence in this age group which may be playing a role in this finding as parents may be more involved in their child’s life at age 18 years than at age 35 years.

Our findings also indicate the importance of examining mobility: there was evidence that downward mobility shows associations with depressive symptoms that are stronger than for stable low education levels, suggesting that being exposed to the process of moving downward in society may also play a role in worse mental health. However, it could also be the reverse with worse mental health being associated with lower subsequent academic attainment [42]. The CLHNS did not collect data on mental health before the children were 18, and therefore it is possible children were experiencing higher levels of mental health problems which led to worse educational achievement. Being downwardly mobile comes with a loss of social status and unexpected changes in socioeconomic circumstances which can be disruptive and stress-inducing [22,43,44]. Lower education may be associated with reduced job prospects and subsequent lower income, which may also play a role in mental ill health. Reduced locus of control for downwardly mobile participants may also contribute to worse mental health, whereas those from persistently lower education and SEP do not feel their circumstances have changed and therefore do not feel they have less control over them [22]. Participants may also feel as though they have not met parental expectations which could contribute to worse mental health [45].

Why we found associations for depression, but not suicidal ideation or psychological distress was not clear. Suicidal ideation can be a more acute and episodic response to an event than a chronic build up of long term social exposures, which may indicate why its presence was less affected by changes in education [46]. Our findings partially replicate findings from an Australian study that found no association with social mobility when using financial status as their indicator, but did find some evidence of associations when using dwelling status [19]. This still differs from our findings as dwelling status, like education, is a more chronic measure of SEP, whereas financial status is more acute. Additionally, stigma towards suicide may partially explain findings, resulting in lower reporting of this outcome, and therefore associations being drawn towards the null. Similarly, the measure of psychological distress encompasses several symptoms of common mental disorders which may not be so susceptible to changes in SEP compared to depression [39]. A wide range of symptoms were assessed that do not lead to one diagnosable health condition, and again may be more easily resolved with changes in situational circumstances. Additionally, our one point-in-time (e.g., last two weeks) measures may not fully capture the episodic or changing mental health symptoms over shorter time periods. It could also be that as this was a secondary analysis of a dataset not designed to answer these questions, the study may simply be underpowered to detect anything other than large differences for these two outcomes. Our findings should be interpreted as exploratory analyses, and additional research is needed to investigate any causal relationships or mechanisms.

### Strengths and limitations

Our study is one of the first to explore associations between educational mobility and mental health in an LMIC. One of the main strengths was the longitudinal study design which mitigates recall bias of parental education, increasing the accuracy of the measure. Similarly, by examining own education at age 35 years, it is likely participants would have achieved their full educational potential, limiting bias in the study, which could be present in the younger age group we analysed. However, our findings of this exploratory analysis should also be interpreted alongside the study’s limitations. Firstly, there is no cut-off to determine higher levels of depressive symptoms in the Filipino population, but we employed a data driven approach used by other studies. Secondly, we only explored one measure of SEP. We considered using other measures but due to seasonal fluctuations in income, a lack of a social class measure appropriate for this setting, and a wealth index more likely to be measuring household SEP, we decided that education was the most applicable. Other measures of SEP may show different associations. Thirdly, we cannot state causality as there may be some unmeasured confounding as we could not control for some variables, e.g., sexual orientation, due to data availability and findings should not be interpreted as such. Finally, there may have been an interaction with sex, however, there were too small numbers in some categories to test this and present stratified results.

## Conclusion

Our findings support an association between downward educational mobility and higher odds of depressive symptoms in adulthood in a LMIC. Associations of downward mobility with higher odds of depressive symptoms and suicidal ideation are seen in late adolescence. Further research is needed to examine why downward mobility leads to greater risk of depressive symptoms.

## Supporting information

S1 Text Additional descriptives and analysis. (DOCX)

S2 Text Age 18 years imputed and complete case analysis. (DOCX)

S3 Text Age 35 years complete case analysis. (DOCX)

## References

[1] GBD 2019 Mental Disorders Collaborators. Global, regional, and national burden of 12 mental disorders in 204 countries and territories, 1990-2019: a systematic analysis for the Global Burden of Disease Study 2019. Lancet Psychiatry. 2022;9(2):137–50. doi: 10.1016/S2215-0366(21)00395-3 35026139 PMC8776563

[2] World Health Organisation. World mental health report: Transforming mental health for all. 2022.

[3] Lund C, Breen A, Flisher AJ, Kakuma R, Corrigall J, Joska JA, et al. Poverty and common mental disorders in low and middle income countries: A systematic review. Soc Sci Med. 2010;71(3):517–28. doi: 10.1016/j.socscimed.2010.04.027 20621748 PMC4991761

[4] Iemmi V, Bantjes J, Coast E, Channer K, Leone T, McDaid D, et al. Suicide and poverty in low-income and middle-income countries: a systematic review. Lancet Psychiatry. 2016;3(8):774–83. doi: 10.1016/S2215-0366(16)30066-9 27475770

[5] Milner A, Page A, LaMontagne AD. Cause and effect in studies on unemployment, mental health and suicide: a meta-analytic and conceptual review. Psychol Med. 2014;44(5):909–17. doi: 10.1017/S0033291713001621 23834819

[6] Lund C, Brooke-Sumner C, Baingana F, Baron EC, Breuer E, Chandra P, et al. Social determinants of mental disorders and the Sustainable Development Goals: a systematic review of reviews. Lancet Psychiatry. 2018;5(4):357–69. doi: 10.1016/S2215-0366(18)30060-9 29580610

[7] Galobardes B, Shaw M, Lawlor DA, Lynch JW, Davey Smith G. Indicators of socioeconomic position (part 1). J Epidemiol Community Health. 2006;60(1):7–12. doi: 10.1136/jech.2004.023531 16361448 PMC2465546

[8] Yen IH, Moss N. Unbundling education: a critical discussion of what education confers and how it lowers risk for disease and death. Ann N Y Acad Sci. 1999;896:350–1. doi: 10.1111/j.1749-6632.1999.tb08138.x 10681919

[9] Ross CE, Mirowsky J. Sex differences in the effect of education on depression: resource multiplication or resource substitution?. Soc Sci Med. 2006;63(5):1400–13. doi: 10.1016/j.socscimed.2006.03.013 16644077

[10] Iversen V, Krishna A, Sen K. Social Mobility in Developing Countries. Oxford University Press 2021. doi: 10.1093/oso/9780192896858.001.0001

[11] Islam S, Jaffee SR. Social mobility and mental health: A systematic review and meta-analysis. Soc Sci Med. 2024;340:116340. doi: 10.1016/j.socscimed.2023.116340 38006845

[12] Lareau A. Cultural Knowledge and Social Inequality. Am Sociol Rev. 2015;80(1):1–27. doi: 10.1177/0003122414565814

[13] Curl H, Lareau A, Wu T. Cultural conflict: The implications of changing dispositions among the upwardly mobile. Sociological Forum. 2018;33(4):877–99.

[14] Friedman S. Habitus Clivé and the Emotional Imprint of Social Mobility. The Sociological Review. 2016;64(1):129–47. doi: 10.1111/1467-954x.12280

[15] Bush NR, Lane RD, McLaughlin KA. Mechanisms Underlying the Association Between Early-Life Adversity and Physical Health: Charting a Course for the Future. Psychosom Med. 2016;78(9):1114–9. doi: 10.1097/PSY.0000000000000421 27763991 PMC5111624

[16] de Quadros L de CM, Quevedo L de A, Motta JV dos S, Carraro A, Ribeiro FG, Horta BL, et al. Social Mobility and Mental Disorders at 30 Years of Age in Participants of the 1982 Cohort, Pelotas, Rio Grande Do Sul - RS. PLoS One. 2015;10(10):e0136886. doi: 10.1371/journal.pone.0136886 26448480 PMC4598184

[17] Palomar-Lever J, Lanzagorta N. Pobreza, recursos psicológicos y movilidad social. Latin American Journal of Psychology. 2005;37:9–45.

[18] GBD 2017 Causes of Death Collaborators. Global, regional, and national age-sex-specific mortality for 282 causes of death in 195 countries and territories, 1980-2013: a systematic analysis for the Global Burden of Disease Study 2017. Lancet. 2018;392(10159):1736–88. doi: 10.1016/S0140-6736(18)32203-7 30496103 PMC6227606

[19] Dal Grande E, Chittleborough CR, Wu J, Shi Z, Goldney RD, Taylor AW. Effect of social mobility in family financial situation and housing tenure on mental health conditions among South Australian adults: results from a population health surveillance system, 2009 to 2011. BMC Public Health. 2015;15:675. doi: 10.1186/s12889-015-2022-9 26184770 PMC4504347

[20] Howe LD, Galobardes B, Matijasevich A, Gordon D, Johnston D, Onwujekwe O, et al. Measuring socio-economic position for epidemiological studies in low- and middle-income countries: a methods of measurement in epidemiology paper. Int J Epidemiol. 2012;41(3):871–86. doi: 10.1093/ije/dys037 22438428 PMC3396323

[21] Ward JB, Haan MN, Garcia ME, Lee A, To TM, Aiello AE. Intergenerational education mobility and depressive symptoms in a population of Mexican origin. Ann Epidemiol. 2016;26(7):461–6. doi: 10.1016/j.annepidem.2016.05.005 27346705 PMC4995110

[22] Gugushvili A, Zhao Y, Bukodi E. “Falling from grace” and “rising from rags”: Intergenerational educational mobility and depressive symptoms. Soc Sci Med. 2019;222:294–304. doi: 10.1016/j.socscimed.2018.12.027 30677643

[23] Tooth L, Mishra G. Intergenerational educational mobility on general mental health and depressive symptoms in young women. Qual Life Res. 2013;22(7):1589–602. doi: 10.1007/s11136-012-0310-8 23138380

[24] World Bank. GDP per capita (current US$) - Philippines 2024. n.d. [cited 2024 March 4]. https://data.worldbank.org/indicator/NY.GDP.PCAP.CD?locations=PH

[25] Philippines Statistics Authority. 2020 Census of Population and Housing: Central Visayas. 2020.

[26] Adair LS, Popkin BM, Akin JS, Guilkey DK, Gultiano S, Borja J, et al. Cohort profile: the Cebu longitudinal health and nutrition survey. Int J Epidemiol. 2011;40(3):619–25. doi: 10.1093/ije/dyq085 20507864 PMC3147061

[27] Radloff LS. The CES-D Scale. Applied Psychological Measurement. 1977;1(3):385–401. doi: 10.1177/014662167700100306

[28] Puyat JH, Gastardo-Conaco MaC, Natividad J, Banal MA. Depressive symptoms among young adults in the Philippines: Results from a nationwide cross-sectional survey. Journal of Affective Disorders Reports. 2021;3:100073. doi: 10.1016/j.jadr.2020.100073

[29] Roberts RE, Andrews JA, Lewinsohn PM, Hops H. Assessment of depression in adolescents using the Center for Epidemiologic Studies Depression Scale. Psychological Assessment: A Journal of Consulting and Clinical Psychology. 1990;2(2):122.

[30] Tran T, Nguyen H, Shochet I, Nguyen N, La N, Wurfl A, et al. Psychometric properties of the centre for epidemiologic studies depression scale revised – vietnamese version (CESDR-V) among adolescents. Psychiatry Research Communications. 2024;4(2):100165. doi: 10.1016/j.psycom.2024.100165

[31] Mazlan NH, Ahmad A. Validation of the Malay-translated version of the Center for Epidemiological Study—Depression Scale (CES-D). ASEAN Journal of Psychiatry. 2014;15(1):54–65.

[32] Mueller Y, Cristofani S, Rodriguez C, Malaguiok RT, Gil T, Grais RF, et al. Integrating mental health into primary care for displaced populations: the experience of Mindanao, Philippines. Confl Health. 2011;5:3. doi: 10.1186/1752-1505-5-3 21385338 PMC3060114

[33] Vizcarra B, Hassan F, Hunter WM, Muñoz SR, Ramiro L, De Paula CS. Partner violence as a risk factor for mental health among women from communities in the Philippines, Egypt, Chile, and India. Inj Control Saf Promot. 2004;11(2):125–9. doi: 10.1080/15660970412331292351 15370349

[34] Varghese JS, Hall RW, Adair LS, Patel SA, Martorell R, Belleza DE, et al. Subjective social status is associated with happiness but not weight status or psychological distress: An analysis of three prospective birth cohorts from low- and middle-income countries. Wellbeing Space Soc. 2022;3:None. doi: 10.1016/j.wss.2022.100115 36518911 PMC9732742

[35] Ganihartono I. Psychiatric morbidity among patients attending the Bangetayu community health centre in Indonesia. Buletin Penelitian Kesehatan. 1996;24(4).

[36] Tuan T, Harpham T, Huong NT. Validity and reliability of the self-reporting questionnaire 20 items in Vietnam. Hong Kong Journal of Psychiatry. 2004;14(3):15.

[37] Graham E, Jordan LP, Yeoh BSA. Parental migration and the mental health of those who stay behind to care for children in South-East Asia. Soc Sci Med. 2015;132:225–35. doi: 10.1016/j.socscimed.2014.10.060 25464878 PMC4405005

[38] Harding TW, de Arango MV, Baltazar J, Climent CE, Ibrahim HH, Ladrido-Ignacio L, et al. Mental disorders in primary health care: a study of their frequency and diagnosis in four developing countries. Psychol Med. 1980;10(2):231–41. doi: 10.1017/s0033291700043993 7384326

[39] Beusenberg M, Orley JH, Organization WH. A User’s guide to the self reporting questionnaire (SRQ). World Health Organization; 1994.

[40] Giang KB, Allebeck P, Kullgren G, Tuan NV. The Vietnamese version of the Self Reporting Questionnaire 20 (SRQ-20) in detecting mental disorders in rural Vietnam: a validation study. Int J Soc Psychiatry. 2006;52(2):175–84. doi: 10.1177/0020764006061251 16615249

[41] van Zwieten A, Dai J, Blyth FM, Wong G, Khalatbari-Soltani S. Overadjustment bias in systematic reviews and meta-analyses of socio-economic inequalities in health: a meta-research scoping review. Int J Epidemiol. 2024;53(1):dyad177. doi: 10.1093/ije/dyad177 38129958 PMC10859162

[42] Riglin L, Petrides KV, Frederickson N, Rice F. The relationship between emotional problems and subsequent school attainment: a meta-analysis. J Adolesc. 2014;37(4):335–46. doi: 10.1016/j.adolescence.2014.02.010 24793380

[43] Stacey B. Some Psychological Consequences of Inter-generation Mobility. Human Relations. 1967;20(1):3–12. doi: 10.1177/001872676702000101

[44] Tiikkaja S, Sandin S, Malki N, Modin B, Sparén P, Hultman CM. Social class, social mobility and risk of psychiatric disorder--a population-based longitudinal study. PLoS One. 2013;8(11):e77975. doi: 10.1371/journal.pone.0077975 24260104 PMC3829839

[45] Iqbal S, Hamdani A, Mazhar S, Munawar A, Tanvir M, Dogar S. Exploring the role of parental expectations and pressures on students’ academic performance and mental health. Migr Lett. 2024;21(S9):1232–42.

[46] Kessler RC, Aguilar-Gaxiola S, Borges G, Chiu WT, Fayyad J, Browne MO. Persistence of suicidal behaviors over time. In: Kessler RC. Suicide: Global perspectives from the WHO World Mental Health Surveys. New York, NY, US: Cambridge University Press; 2012. 75–85.

