# Supplementary material for: Intergenerational educational mobility and mental health: Evidence from a Filipino birth cohort
Source: PLOS Glob Public Health. 2025 Aug 12;5(8):e0004570. doi: 10.1371/journal.pgph.0004570 (PMC12342240; doi:10.1371/journal.pgph.0004570)
Supplement: S1 Text — (DOCX) [file pgph.0004570.s001.docx]

Table A. Summary statistics of imputed and complete case datasets at age 18 and age 35 years

|  | |  |  |  |  |
| --- | --- | --- | --- | --- | --- |
| Variable | | **% Imputed*** |  | **Complete Case 2002 (Age 18 years)**  n (%)  n = 2020 | **Complete Case 2018 (Age 35 years)**  n (%)  n = 1193 |
| Male | | . |  | 1071 (53.0) | 640 (53.7) |
| Urban | | . |  | 1491 (73.8) | 876 (73.4) |
| Own education  High  Low | | 35.3 |  | 1184 (58.6)  836 (41.2) | 832 (69.7)  361 (30.3) |
| Parental education  High  Low | | . |  | 695 (34.4)  1325 (65.6) | 355 (29.8)  838 (70.2) |
| Mobility  Stable high  Upward  Stable low  Downward | | 35.3 |  | 538 (26.6)  646 (32.0)  679 (33.6)  157 (7.8) | 310 (26.0)  522 (43.8)  316 (26.5)  45 (3.8) |
| High depressive symptoms | | 41.2 |  | 289 (14.3) | 99 (8.3) |
| Suicidal ideation | | 41.2 |  | 325 (16.1) | 89 (7.5) |
| Psychological distress | | 35.0 |  | - | 165 (13.8) |
|  | *Figures shown for 2018 imputation. ‘.‘ = no imputation required | | | | |

Table B. Summary statistics of age 35 years imputed data, stratified by educational mobility

|  | Stable high | Upward mobility | | Downward mobility | | Stable low |
| --- | --- | --- | --- | --- | --- | --- |
| Urban | 86.8 | 69.0 | 84.8 | | 63.6 | |
| Female | 46.3 | 56.1 | 32.9 | | 33.9 | |
| Depressive symptoms | 7.4 | 6.6 | 20.0 | | 10.3 | |
| Suicidal ideation | 7.0 | 6.5 | 10.6 | | 9.1 | |
| Psychological distress | 13.6 | 11.6 | 21.3 | | 14.0 | |

Table C. Unadjusted odds ratios of association between education levels and mental health outcomes at age 35 years (imputed)

| Variable | Depressive symptoms | | | | Suicidal ideation | | | Psychological distress | | |
| --- | --- | --- | --- | --- | --- | --- | --- | --- | --- | --- |
|  | **N** | **OR***^1^* | **95% CI***^1^* | **p-value** | **OR***^1^* | **95% CI***^1^* | **p-value** | **OR***^1^* | **95% CI***^1^* | **p-value** |
| Depressive Symptoms | | | | | | | |  |  |  |
| Parental education at birth | 2,038 |  |  |  |  |  |  |  |  |  |
| High |  | 1.00 | Ref |  | 1.00 | Ref |  | 1.00 | Ref |  |
| Low |  | 0.90 | 0.59, 1.38 | 0.634 | 1.01 | 0.63, 1.64 | 0.953 | 0.84 | 0.61, 1.16 | 0.294 |
| Own education age 35 years |  |  |  |  |  |  |  |  |  |  |
| High |  | 1.00 | Ref |  | 1.00 | Ref |  | 1.00 | Ref |  |
| Low |  | 1.77 | 1.15, 2.72 | 0.010 | 1.42 | 0.91, 2.23 | 0.124 | 1.25 | 0.89, 1.75 | 0.195 |
| *^1^* OR = Odds Ratio, CI = Confidence Interval | | | | | | | |  |  |  |

Table D. Association between educational mobility and mental health outcomes at age 35 years (2018)

| Variable | Unadjusted | | | | Adjusted* | | | |
| --- | --- | --- | --- | --- | --- | --- | --- | --- |
|  | **N** | **OR***^1^* | **95% CI***^1^* | **p-value** | | **OR***^1^* | **95% CI***^1^* | **p-value** |
| Depressive Symptoms | | | | | | | | |
| Educational Mobility age 35 years | 2,038 |  |  |  | |  |  |  |
| Stable High |  | 1.00 | Ref |  | | 1.00 | Ref |  |
| Downward |  | 3.14 | 1.47, 6.74 | 0.003 | | 2.98 | 1.38, 6.44 | 0.005 |
| Stable Low |  | 1.44 | 0.83, 2.55 | 0.196 | | 1.38 | 0.78, 2.46 | 0.269 |
| Upward |  | 0.90 | 0.54, 1.52 | 0.694 | | 0.95 | 0.56, 1.61 | 0.839 |
| Suicidal Ideation | | | | | | | | |
| Stable High |  | 1.00 | Ref |  | | 1.00 | Ref |  |
| Downward |  | 1.57 | 0.58, 4.17 | 0.369 | | 1.53 | 0.57, 4.08 | 0.397 |
| Stable Low |  | 1.34 | 0.73, 2.45 | 0.342 | | 1.34 | 0.72, 2.49 | 0.360 |
| Upward |  | 0.94 | 0.54, 1.63 | 0.819 | | 0.97 | 0.55, 1.71 | 0.921 |
| Psychological Distress | | | | | | | | |
| Stable High |  | 1.00 | Ref |  | | 1.00 | Ref |  |
| Downward |  | 1.71 | 0.84, 3.46 | 0.137 | | 1.96 | 0.95, 4.04 | 0.068 |
| Stable Low |  | 1.04 | 0.68, 1.59 | 0.856 | | 1.28 | 0.83, 1.98 | 0.271 |
| Upward |  | 0.83 | 0.58, 1.20 | 0.328 | | 0.83 | 0.57, 1.21 | 0.331 |
| *^1^* OR = Odds Ratio, CI = Confidence Interval  *Adjusted for sex and urbanicity at birth | | | | | | | | |
