# Supplementary material for: Intergenerational educational mobility and mental health: Evidence from a Filipino birth cohort
Source: PLOS Glob Public Health. 2025 Aug 12;5(8):e0004570. doi: 10.1371/journal.pgph.0004570 (PMC12342240; doi:10.1371/journal.pgph.0004570)
Supplement: S2 Text — (DOCX) [file pgph.0004570.s002.docx]

**Age 18 Years Imputed Analysis**

There is statistical evidence to suggest that low parental education, and low own education is associated with depressive symptoms and suicidal ideation at age 18 years. The association persists after adjustment for sex and urbanicity, although CI are close to/cross the null for parental education and suicidal ideation.

Table A. Association between education levels and mental health outcomes using age 18 years imputed dataset

| Variable | | | Unadjusted | | | | | | | | Adjusted* | | | | | | | |  |  |
| --- | --- | --- | --- | --- | --- | --- | --- | --- | --- | --- | --- | --- | --- | --- | --- | --- | --- | --- | --- | --- |
|  |  |  | **N** | | **OR***^1^* | | | **95% CI***^1^* | **p-value** | | | **OR***^1^* | | **95% CI***^1^* | | **p-value** | | |  |  |
| Depressive Symptoms | | | | | | | | | | | | | | | | | | | |  |
| Parental education at birth | 2,038 | | | | |  | |  | |  | | |  | |  | |  | | |  |
| High |  | | | | | 1.00 | | Ref | |  | | | 1.00 | | Ref | |  | | |  |
| Low |  | | | | | 1.54 | | 1.17, 2.04 | | 0.002 | | | 1.50 | | 1.13, 1.99 | | 0.006 | | |  |
| Own education | | 2,038 | |  | | |  | | |  | | |  | |  | | |  | | |
| High | |  | | 1.00 | | | Ref | | |  | | | 1.00 | | Ref | | |  | | |
| Low | |  | | 1.98 | | | 1.54, 2.54 | | | <0.001 | | | 2.25 | | 1.73, 2.92 | | | <0.001 | | |
| Suicidal Ideation | | | | | | | | | | | | | | | | | | | |  |
| Parental education at birth | | 2,038 | |  | | |  | | |  | | |  | |  | | |  | | |
| High | |  | | 1.00 | | | Ref | | |  | | | 1.00 | | Ref | | |  | | |
| Low | |  | | 1.29 | | | 1.00, 1.67 | | | 0.049 | | | 1.27 | | 0.97, 1.65 | | | 0.077 | | |
| Own education | | 2,038 | |  | | |  | | |  | | |  | |  | | |  | | |
| High | |  | | 1.00 | | | Ref | | |  | | | 1.00 | | Ref | | |  | | |
| Low | |  | | 1.29 | | | 1.02, 1.64 | | | 0.035 | | | 1.51 | | 1.18, 1.94 | | | 0.001 | | |

When exploring the effect of educational mobility in the whole imputed sample, evidence suggests that for depressive symptoms, being downwardly mobile or remaining in the lower education category results in higher odds of depressive symptoms. Whilst point estimates for upward mobility suggested increased odds compared to participant in stable-high, there was little evidence of this association. For suicidal ideation, only those remaining in the stable-low category were at higher odds than participants in the stable-high category, with the association remaining after adjustment for sex and urbanicity. There was no evidence of associations in the other two categories, compared to stable-high.

Table B. Association between educational mobility and mental health outcomes using age 18 years imputed dataset

| Variable | Unadjusted | | | | Adjusted* | | | |
| --- | --- | --- | --- | --- | --- | --- | --- | --- |
|  | **N** | **OR***^1^* | **95% CI***^1^* | **p-value** | | **OR***^1^* | **95% CI***^1^* | **p-value** |
| Educational Mobility | 2,038 |  |  |  | |  |  |  |
| Depressive Symptoms | | | | | | | | |
| Stable High |  | 1.00 | Ref |  | | 1.00 | Ref |  |
| Downward |  | 1.78 | 1.06, 2.97 | 0.029 | | 2.02 | 1.20, 3.41 | 0.008 |
| Stable Low |  | 2.36 | 1.68, 3.33 | <0.001 | | 2.54 | 1.79, 3.61 | <0.001 |
| Upward |  | 1.26 | 0.87, 1.82 | 0.230 | | 1.16 | 0.79, 1.69 | 0.452 |
| Suicidal Ideation | | | | | | | | |
| Stable High |  | 1.00 | Ref |  | | 1.00 | Ref |  |
| Downward |  | 1.17 | 0.71, 1.92 | 0.549 | | 1.36 | 0.82, 2.25 | 0.240 |
| Stable Low |  | 1.49 | 1.09, 2.04 | 0.013 | | 1.65 | 1.20, 2.28 | 0.002 |
| Upward |  | 1.19 | 0.86, 1.65 | 0.288 | | 1.10 | 0.79, 1.54 | 0.570 |

**Age 18 years Complete Case Analysis**

There was strong evidence of an association between lower parental education levels and higher odds of depressive symptoms with 50% increased odds in this category. The association was not present after adjustment for sex and urbanicity for suicidal ideation. When exploring own education, there was strong evidence of lower education being associated with higher odds of both outcomes, with the magnitude of effect being greater for depressive symptoms.

Table C. Association between education levels and mental health outcomes using age 18 years complete case dataset

| Variable | | | Unadjusted | | | | | | | | Adjusted* | | | | | | | |  |  |
| --- | --- | --- | --- | --- | --- | --- | --- | --- | --- | --- | --- | --- | --- | --- | --- | --- | --- | --- | --- | --- |
|  |  |  | **N** | | **OR***^1^* | | | **95% CI***^1^* | **p-value** | | | **OR***^1^* | | **95% CI***^1^* | | **p-value** | | |  |  |
| Depressive Symptoms | | | | | | | | | | | | | | | | | | | |  |
| Parental education at birth | 2,027 | | | | |  | |  | |  | | |  | |  | |  | | |  |
| High |  | | | | | 1.00 | | Ref | |  | | | 1.00 | | Ref | |  | | |  |
| Low |  | | | | | 1.54 | | 1.17, 2.04 | | 0.002 | | | 1.50 | | 1.13, 2.00 | | 0.005 | | |  |
| Own education | | 2,020 | |  | | |  | | |  | | |  | |  | | |  | | |
| High | |  | | 1.00 | | | Ref | | |  | | | 1.00 | | Ref | | |  | | |
| Low | |  | | 1.97 | | | 1.53, 2.53 | | | <0.001 | | | 2.25 | | 1.73, 2.92 | | | <0.001 | | |
| Suicidal Ideation | | | | | | | | | | | | | | | | | | | |  |
| Parental education at birth | | 2,027 | |  | | |  | | |  | | |  | |  | | |  | | |
| High | |  | | 1.00 | | | Ref | | |  | | | 1.00 | | Ref | | |  | | |
| Low | |  | | 1.29 | | | 1.00, 1.67 | | | 0.050 | | | 1.27 | | 0.97, 1.65 | | | 0.078 | | |
| Own education | | 2,020 | |  | | |  | | |  | | |  | |  | | |  | | |
| High | |  | | 1.00 | | | Ref | | |  | | | 1.00 | | Ref | | |  | | |
| Low | |  | | 1.30 | | | 1.02, 1.65 | | | 0.032 | | | 1.53 | | 1.19, 1.96 | | | 0.001 | | |

Remaining in low education was associated with higher odds of both depressive symptoms and suicidal ideation. There was strong evidence for this association with depressive symptoms and experiencing downward educational mobility, however, for suicidal ideation, confidence intervals crossed the null indicating no evidence of an association. There was no evidence of an association with either outcome in participants who experienced upward mobility between parental education and own education.

Table D. Association between educational mobility and mental health outcomes using age 18 years complete case dataset

| Variable | Univariate | | | | Multivariable* | | | |
| --- | --- | --- | --- | --- | --- | --- | --- | --- |
|  | **N** | **OR***^1^* | **95% CI***^1^* | **p-value** | | **OR***^1^* | **95% CI***^1^* | **p-value** |
| Educational Mobility | 2,027 |  |  |  | |  |  |  |
| Depressive Symptoms | | | | | | | | |
| Stable High |  | 1.00 | Ref |  | | 1.00 | Ref |  |
| Downward |  | 1.77 | 1.06, 2.96 | 0.030 | | 2.03 | 1.20, 3.41 | 0.008 |
| Stable Low |  | 2.34 | 1.66, 3.30 | <0.001 | | 2.52 | 1.77, 3.57 | <0.001 |
| Upward |  | 1.25 | 0.86, 1.81 | 0.247 | | 1.14 | 0.78, 1.67 | 0.487 |
| Suicidal Ideation | | | | | | | | |
| Stable High |  | 1.00 | Ref |  | | 1.00 | Ref |  |
| Downward |  | 1.17 | 0.71, 1.93 | 0.543 | | 1.37 | 0.82, 2.27 | 0.227 |
| Stable Low |  | 1.50 | 1.09, 2.06 | 0.011 | | 1.67 | 1.21, 2.31 | 0.002 |
| Upward |  | 1.20 | 0.87, 1.66 | 0.275 | | 1.00 | 0.79, 1.54 | 0.559 |
