# Supplementary material for: Intergenerational educational mobility and mental health: Evidence from a Filipino birth cohort
Source: PLOS Glob Public Health. 2025 Aug 12;5(8):e0004570. doi: 10.1371/journal.pgph.0004570 (PMC12342240; doi:10.1371/journal.pgph.0004570)
Supplement: S3 Text — (DOCX) [file pgph.0004570.s003.docx]

**Age 35 Years Complete Case Analysis**

There was no evidence of an association between parental education and any mental health outcome at age 35 years. However, low education in the individual was associated with higher odds of depressive symptoms and psychological distress, in adjusted analysis at age 35 years. There was no evidence of an association with own education and suicidal ideation.

Table A. Association between education levels and mental health outcomes using age 35 years complete case dataset

| Variable | Unadjusted | | | | Adjusted* | | |
| --- | --- | --- | --- | --- | --- | --- | --- |
|  | **N** | **OR***^1^* | **95% CI***^1^* | **p-value** | **OR***^1^* | **95% CI***^1^* | **p-value** |
| Depressive Symptoms | | | | | | | |
| Parental education at birth | 1,193 |  |  |  |  |  |  |
| High |  | 1.00 | Ref |  | 1.00 | Ref |  |
| Low |  | 0.88 | 0.56, 1.36 | 0.560 | 0.91 | 0.58, 1.44 | 0.696 |
| Own education 2018 | 1,193 |  |  |  |  |  |  |
| High |  | 1.00 | Ref |  | 1.00 | Ref |  |
| Low |  | 1.79 | 1.18, 2.72 | 0.006 | 1.68 | 1.10, 2.58 | 0.017 |
| Suicidal Ideation | | | | | | | |
| Parental education at birth | 1,193 |  |  |  |  |  |  |
| High |  | 1.00 | Ref |  | 1.00 | Ref |  |
| Low |  | 1.03 | 0.64, 1.65 | 0.907 | 1.07 | 0.66, 1.74 | 0.787 |
| Own education 2018 | 1,193 |  |  |  |  |  |  |
| High |  | 1.00 | Ref |  | 1.00 | Ref |  |
| Low |  | 1.47 | 0.94, 2.30 | 0.092 | 1.43 | 0.91, 2.26 | 0.112 |
| Psychological Distress | | | | | | | |
| Parental education at birth | 1,193 |  |  |  |  |  |  |
| High |  | 1.00 | Ref |  | 1.00 | Ref |  |
| Low |  | 0.85 | 0.60, 1.21 | 0.369 | 0.87 | 0.61, 1.25 | 0.450 |
| Own education 2018 | 1,193 |  |  |  |  |  |  |
| High |  | 1.00 | Ref |  | 1.00 | Ref |  |
| Low |  | 1.30 | 0.92, 1.83 | 0.169 | 1.54 | 1.08, 2.21 | 0.017 |
| *^1^* OR = Odds Ratio, CI = Confidence Interval  *Adjusted for sex and urbanicity at birth | | | | | | | |

There was strong evidence of an association between downward mobility and higher levels of depressive symptoms at age 35 years. In the adjusted analysis, participants who had downward mobility trajectories are 4.43 times more likely to experience higher levels of depressive symptoms than participants who have consistently high education. Similarly, after adjustment for covariates, there was some statistical evidence of downward mobility being associated with increased odds of experiencing psychological distress. There was no evidence of any association between upward mobility or remaining in the low education category and an association with mental health, compared to remaining in the higher education category.

Table B. Association between educational mobility and mental health outcomes using age 35 years complete case dataset

| Variable | Unadjusted | | | | Adjusted* | | | |
| --- | --- | --- | --- | --- | --- | --- | --- | --- |
|  | **N** | **OR***^1^* | **95% CI***^1^* | **p-value** | | **OR***^1^* | **95% CI***^1^* | **p-value** |
| Depressive Symptoms | | | | | | | | |
| Educational Mobility 2018 | 1,193 |  |  |  | |  |  |  |
| Stable High |  | 1.00 | Ref |  | | 1.00 | Ref |  |
| Downward |  | 4.45 | 1.98, 10.02 | <0.001 | | 4.35 | 1.92, 9.84 | <0.001 |
| Stable Low |  | 1.50 | 0.84, 2.67 | 0.171 | | 1.47 | 0.81, 2.65 | 0.201 |
| Upward |  | 1.02 | 0.58, 1.78 | 0.946 | | 1.11 | 0.63, 1.95 | 0.727 |
| Suicidal Ideation | | | | | | | | |
| Stable High |  | 1.00 | Ref |  | | 1.00 | Ref |  |
| Downward |  | 1.72 | 0.61, 4.82 | 0.141 | | 1.70 | 0.61, 4.77 | 0.313 |
| Stable Low |  | 1.39 | 0.77, 2.50 | 0.295 | | 1.41 | 0.77, 2.56 | 0.263 |
| Upward |  | 0.96 | 0.55, 1.68 | 0.884 | | 1.01 | 0.57, 1.80 | 0.963 |
| Psychological Distress | | | | | | | | |
| Stable High |  | 1.00 | Ref |  | | 1.00 | Ref |  |
| Downward |  | 2.01 | 0.95, 4.26 | 0.069 | | 2.22 | 1.03, 4.77 | 0.042 |
| Stable Low |  | 1.08 | 0.69, 1.70 | 0.721 | | 1.27 | 0.80, 2.01 | 0.309 |
| Upward |  | 0.87 | 0.57, 1.31 | 0.503 | | 0.84 | 0.55, 1.28 | 0.409 |
| *^1^* OR = Odds Ratio, CI = Confidence Interval  *Adjusted for sex and urbanicity at birth | | | | | | | | |
